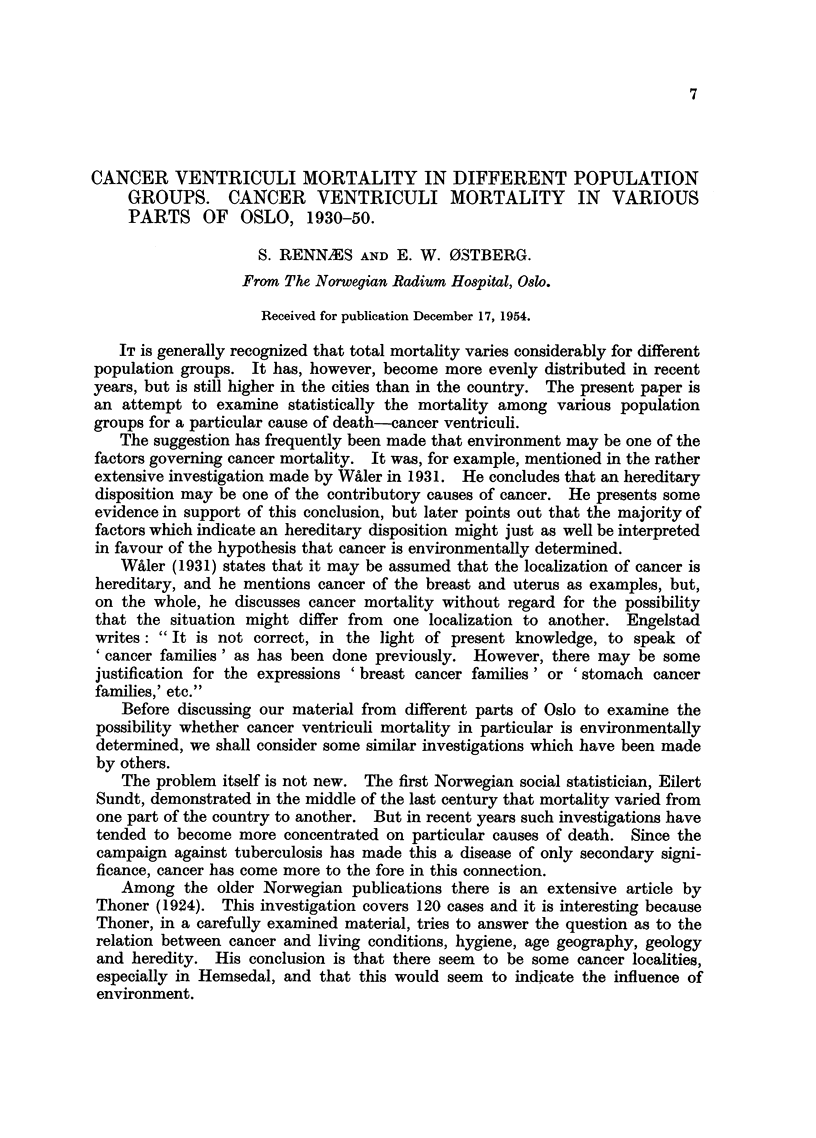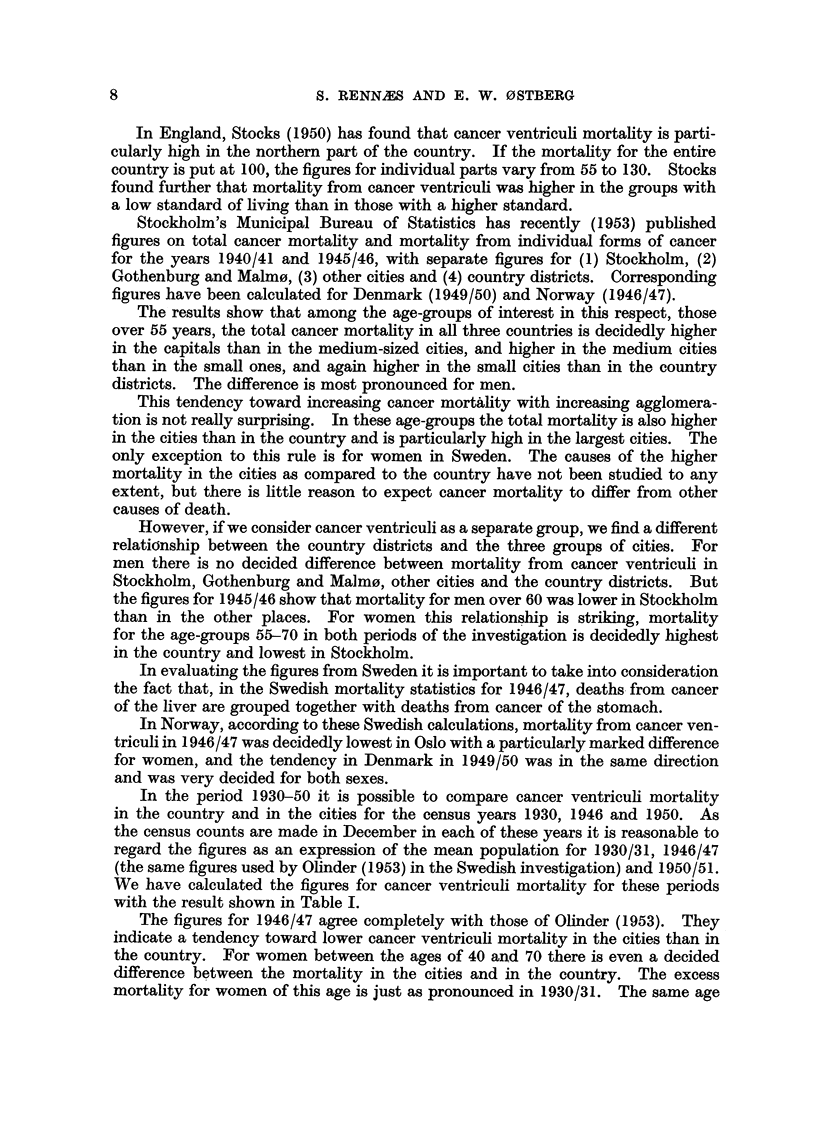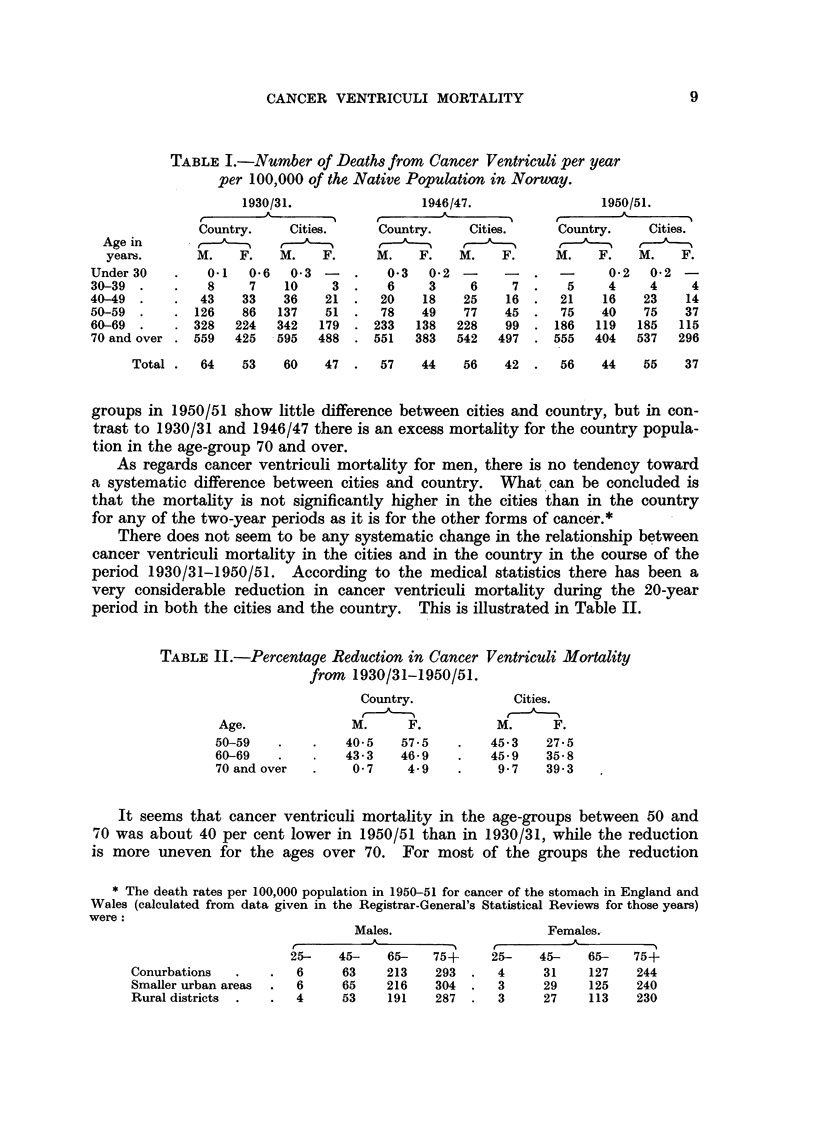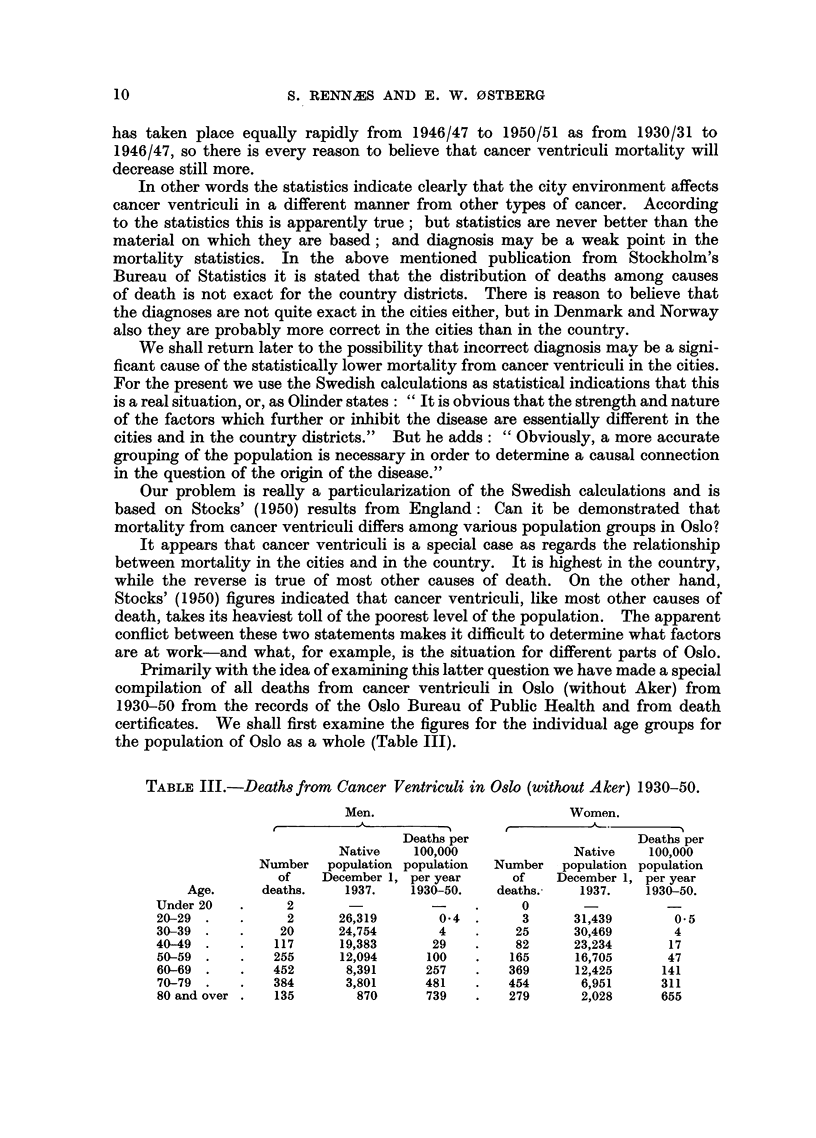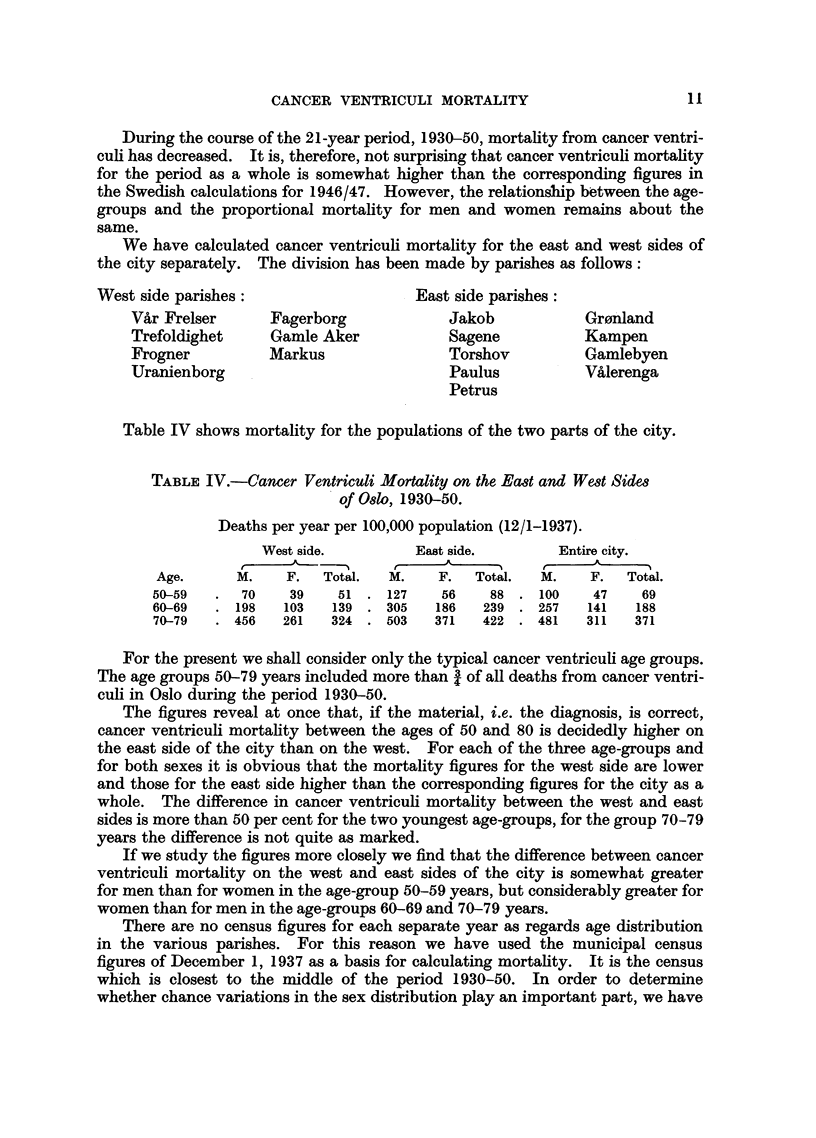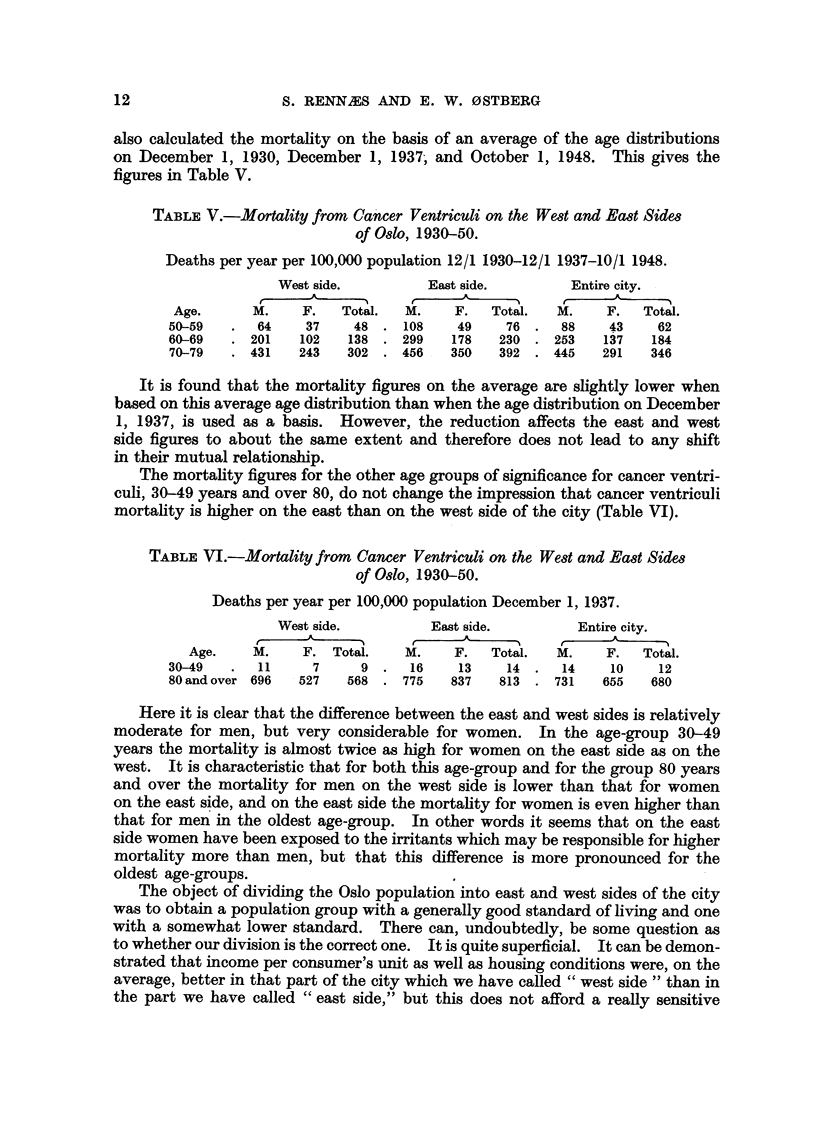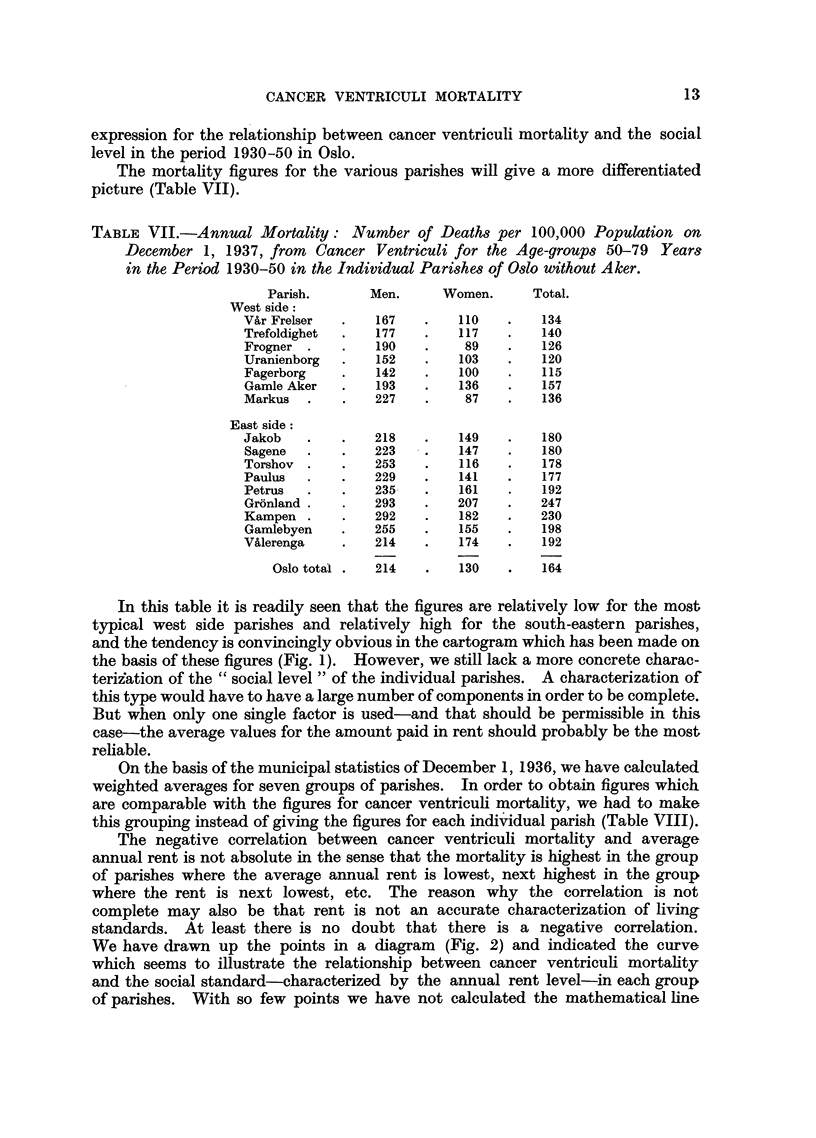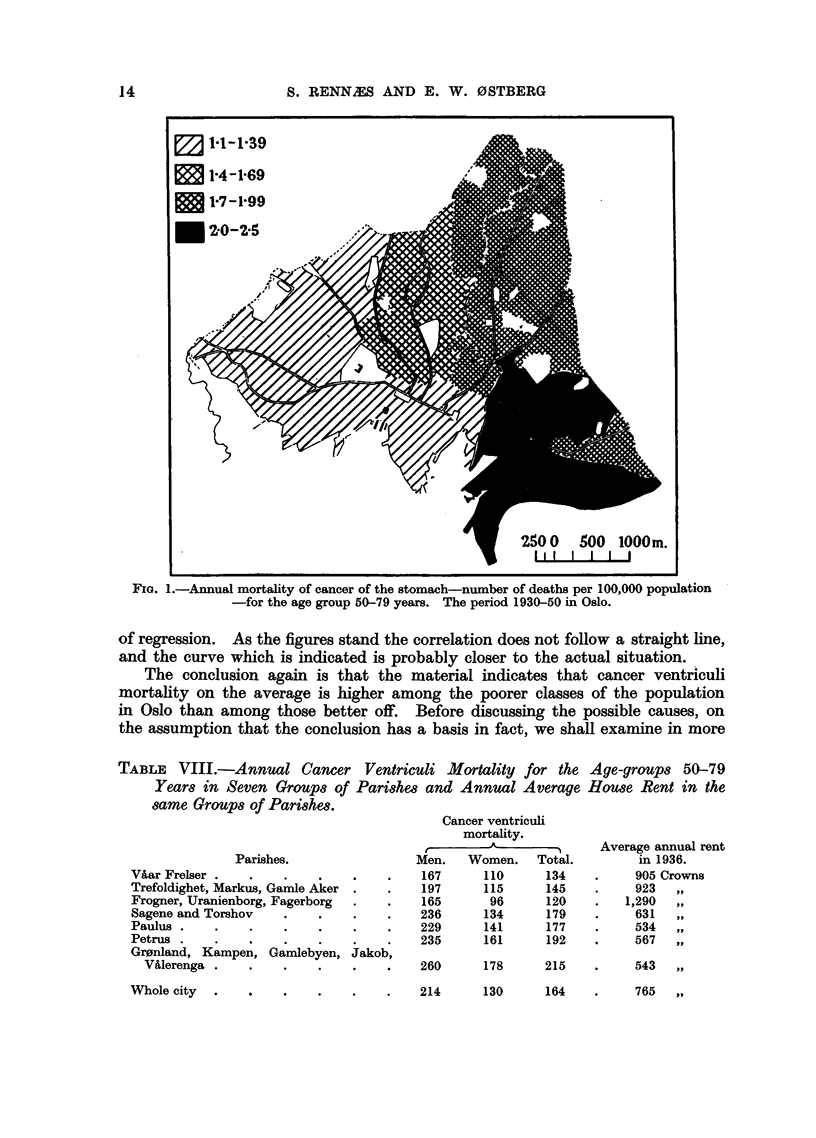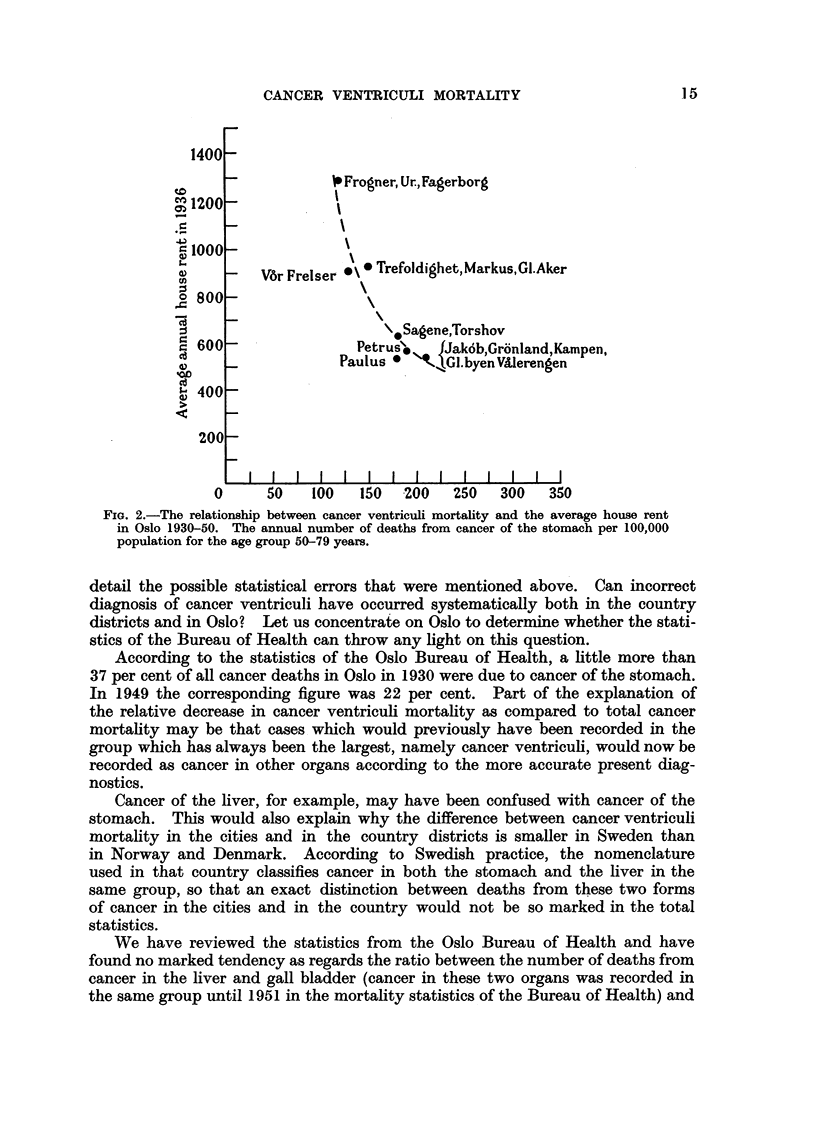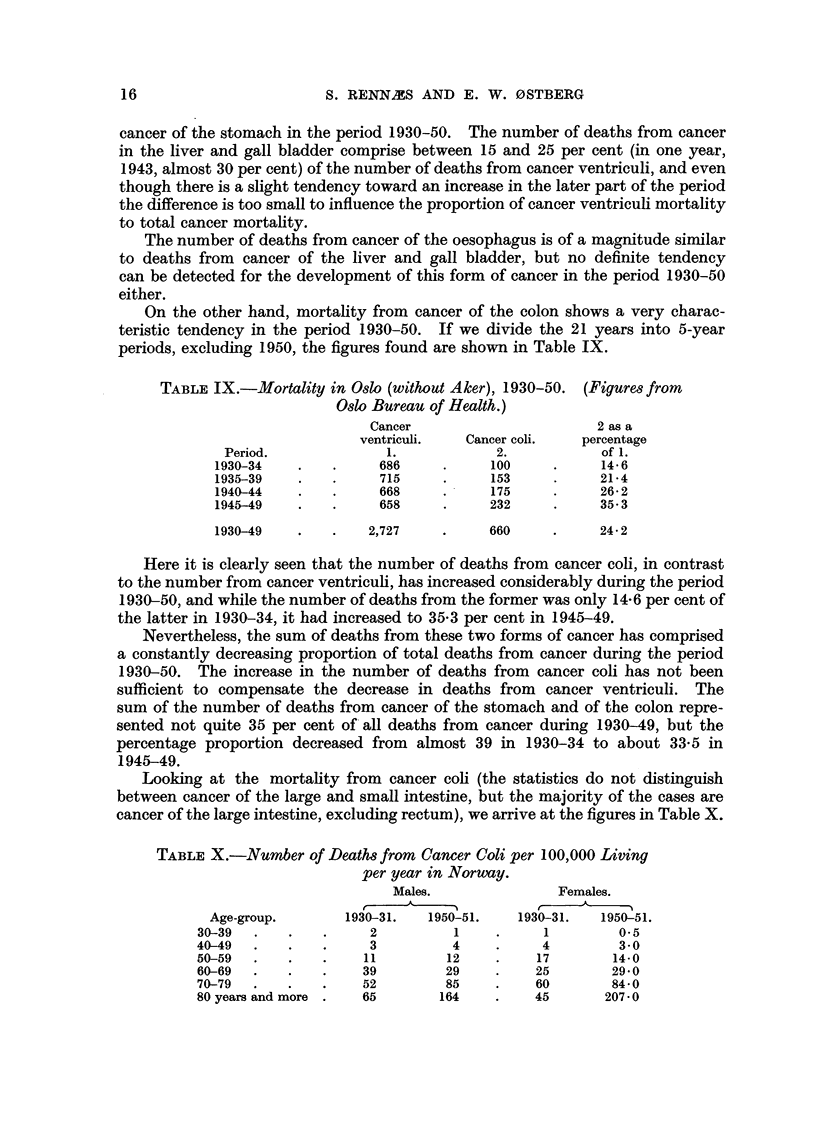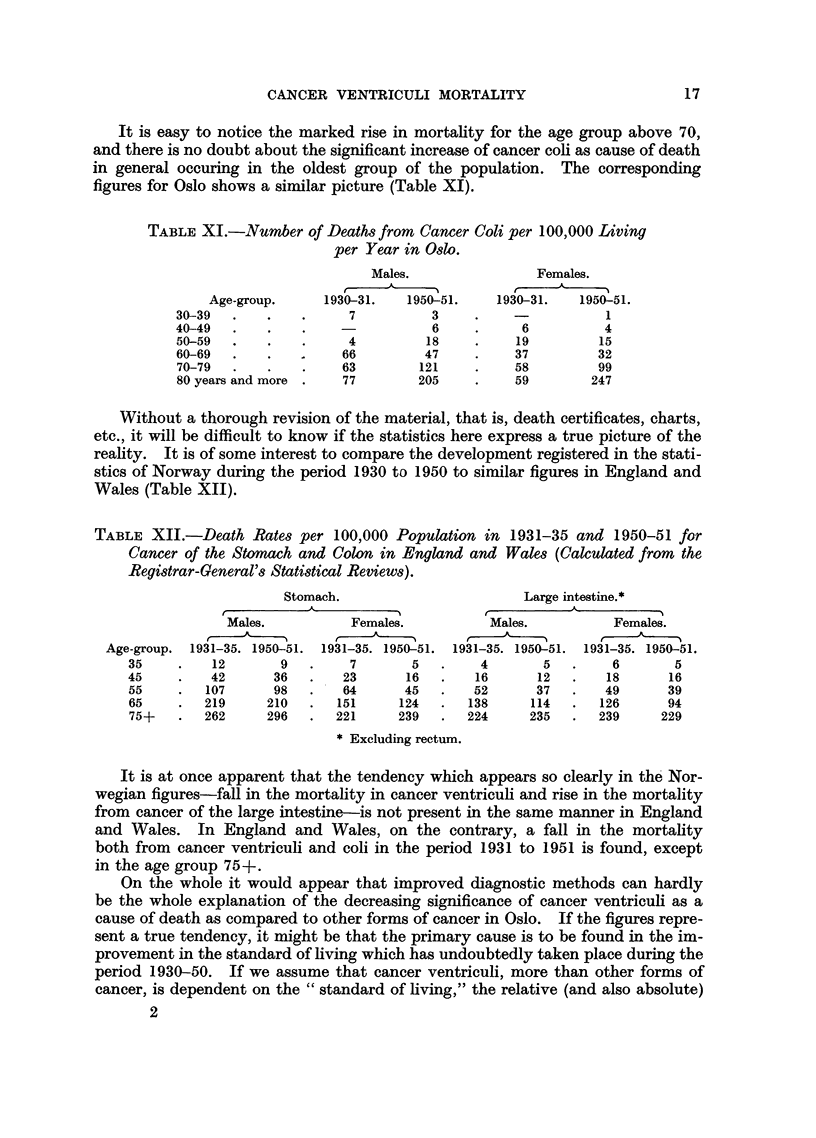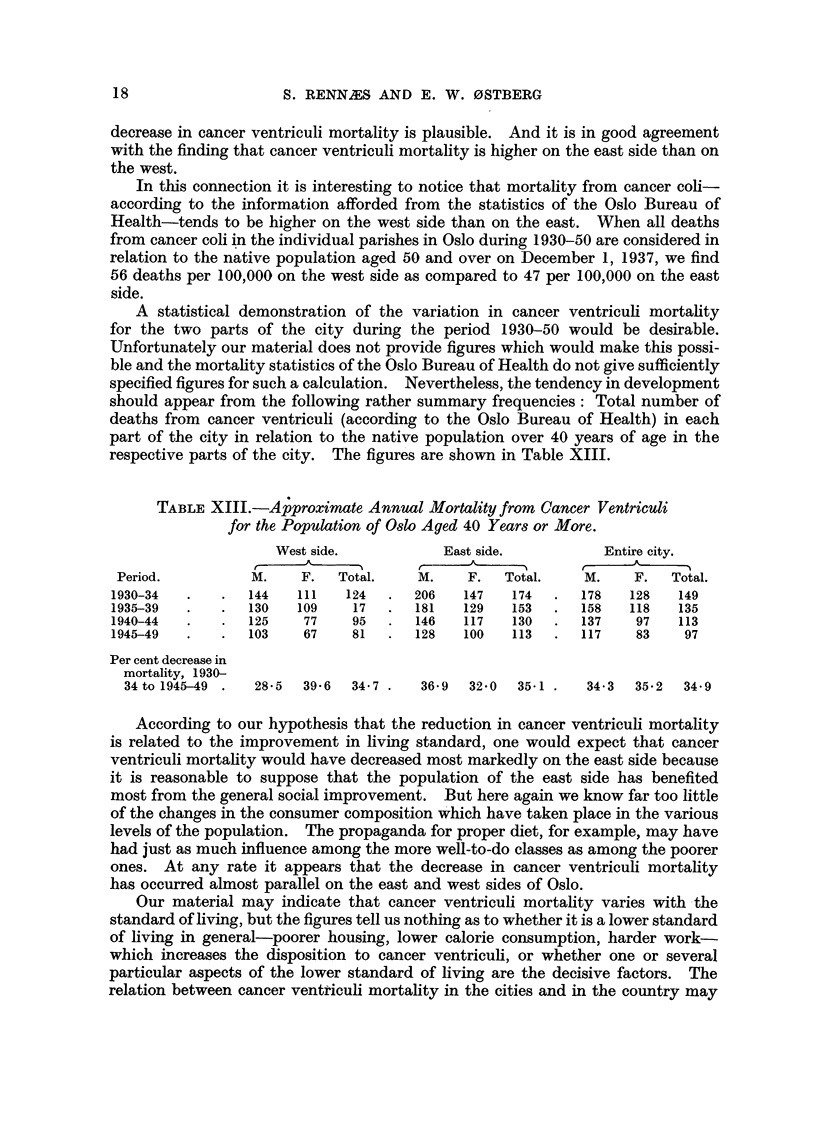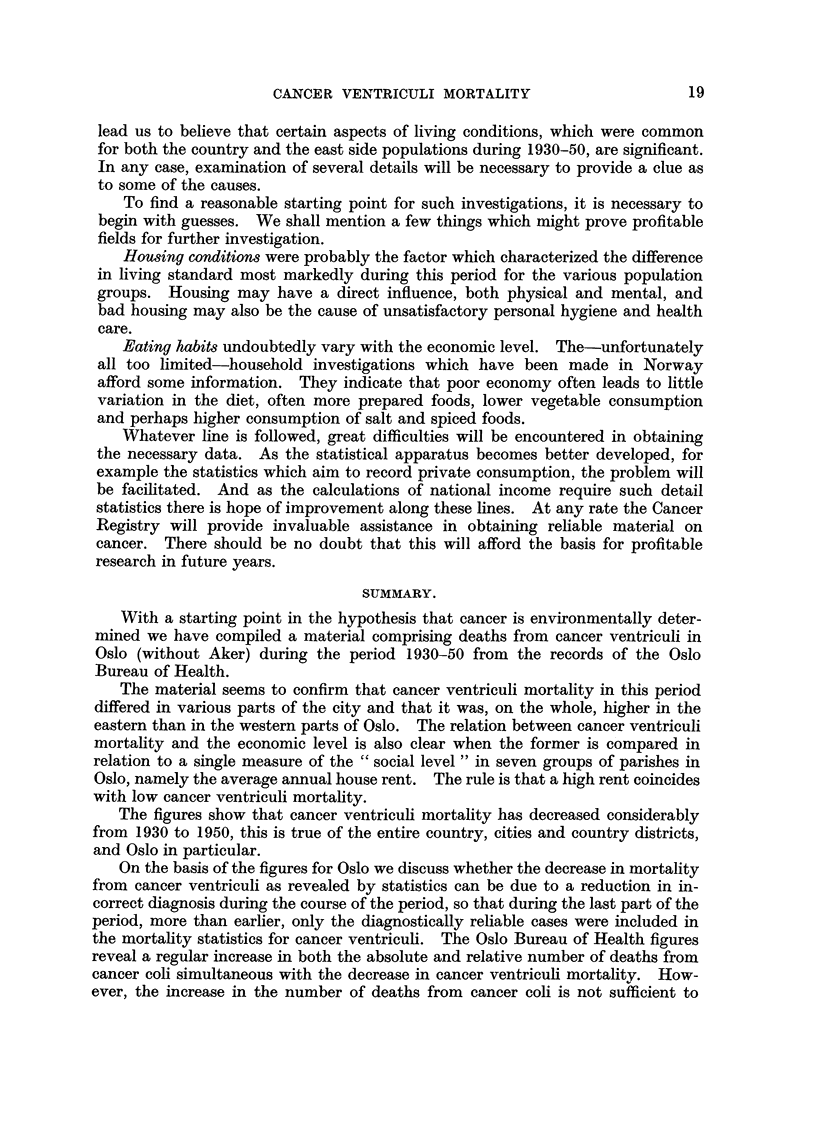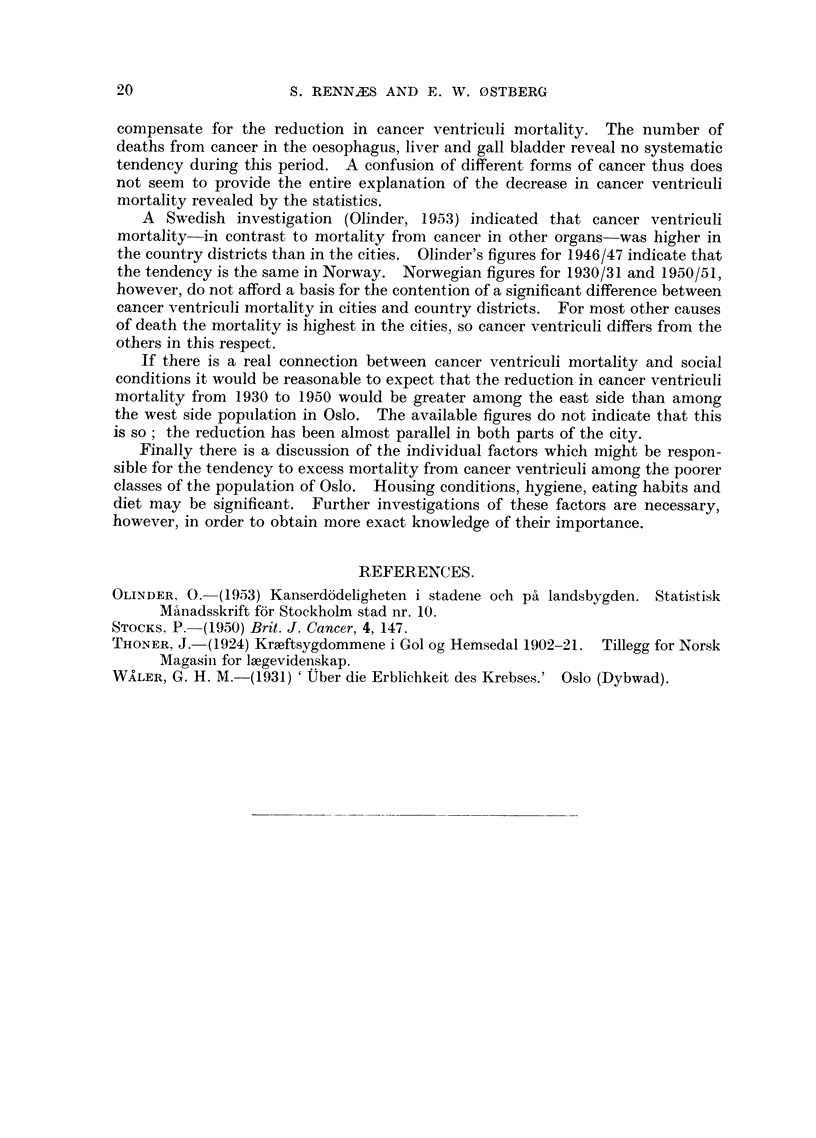# Cancer Ventriculi Mortality in Different Population Groups. Cancer Ventriculi Mortality in Various Parts of Oslo, 1930-50

**DOI:** 10.1038/bjc.1955.2

**Published:** 1955-03

**Authors:** S. Rennæs, E. W. Østberg


### CANCER VENTRICULI MORTALITY IN DIFFERENT POPULATION GROUPS. CANCER VENTRICULI MORTALITY IN VARIOUS PARTS OF OSLO, 1930–50.

S. RENNÆS AND E. W. ØSTBERG.

*From The Norwegian Radium Hospital, Oslo.*

Received for publication December 17, 1954.

It is generally recognized that total mortality varies considerably for different population groups. It has, however, become more evenly distributed in recent years, but is still higher in the cities than in the country. The present paper is an attempt to examine statistically the mortality among various population groups for a particular cause of death—cancer ventriculi.

The suggestion has frequently been made that environment may be one of the factors governing cancer mortality. It was, for example, mentioned in the rather extensive investigation made by Wåler in 1931. He concludes that an hereditary disposition may be one of the contributory causes of cancer. He presents some evidence in support of this conclusion, but later points out that the majority of factors which indicate an hereditary disposition might just as well be interpreted in favour of the hypothesis that cancer is environmentally determined.

Wåler (1931) states that it may be assumed that the localization of cancer is hereditary, and he mentions cancer of the breast and uterus as examples, but, on the whole, he discusses cancer mortality without regard for the possibility that the situation might differ from one localization to another. Engelstad writes : " It is not correct, in the light of present knowledge, to speak of ' cancer families ' as has been done previously. However, there may be some justification for the expressions ' breast cancer families ' or ' stomach cancer families,' etc."

Before discussing our material from different parts of Oslo to examine the possibility whether cancer ventriculi mortality in particular is environmentally determined, we shall consider some similar investigations which have been made by others.

The problem itself is not new. The first Norwegian social statistician, Eilert Sundt, demonstrated in the middle of the last century that mortality varied from one part of the country to another. But in recent years such investigations have tended to become more concentrated on particular causes of death. Since the campaign against tuberculosis has made this a disease of only secondary significance, cancer has come more to the fore in this connection.

Among the older Norwegian publications there is an extensive article by Thoner (1924). This investigation covers 120 cases and it is interesting because Thoner, in a carefully examined material, tries to answer the question as to the relation between cancer and living conditions, hygiene, age geography, geology and heredity. His conclusion is that there seem to be some cancer localities, especially in Hemsedal, and that this would seem to indicate the influence of environment.

In England, Stocks (1950) has found that cancer ventriculi mortality is particularly high in the northern part of the country. If the mortality for the entire country is put at 100, the figures for individual parts vary from 55 to 130. Stocks found further that mortality from cancer ventriculi was higher in the groups with a low standard of living than in those with a higher standard.

Stockholm's Municipal Bureau of Statistics has recently (1953) published figures on total cancer mortality and mortality from individual forms of cancer for the years 1940/41 and 1945/46, with separate figures for (1) Stockholm, (2) Gothenburg and Malmø, (3) other cities and (4) country districts. Corresponding figures have been calculated for Denmark (1949/50) and Norway (1946/47).

The results show that among the age-groups of interest in this respect, those over 55 years, the total cancer mortality in all three countries is decidedly higher in the capitals than in the medium-sized cities, and higher in the medium cities than in the small ones, and again higher in the small cities than in the country districts. The difference is most pronounced for men.

This tendency toward increasing cancer mortality with increasing agglomeration is not really surprising. In these age-groups the total mortality is also higher in the cities than in the country and is particularly high in the largest cities. The only exception to this rule is for women in Sweden. The causes of the higher mortality in the cities as compared to the country have not been studied to any extent, but there is little reason to expect cancer mortality to differ from other causes of death.

However, if we consider cancer ventriculi as a separate group, we find a different relationship between the country districts and the three groups of cities. For men there is no decided difference between mortality from cancer ventriculi in Stockholm, Gothenburg and Malmø, other cities and the country districts. But the figures for 1945/46 show that mortality for men over 60 was lower in Stockholm than in the other places. For women this relationship is striking, mortality for the age-groups 55–70 in both periods of the investigation is decidedly highest in the country and lowest in Stockholm.

In evaluating the figures from Sweden it is important to take into consideration the fact that, in the Swedish mortality statistics for 1946/47, deaths from cancer of the liver are grouped together with deaths from cancer of the stomach.

In Norway, according to these Swedish calculations, mortality from cancer ventriculi in 1946/47 was decidedly lowest in Oslo with a particularly marked difference for women, and the tendency in Denmark in 1949/50 was in the same direction and was very decided for both sexes.

In the period 1930–50 it is possible to compare cancer ventriculi mortality in the country and in the cities for the census years 1930, 1946 and 1950. As the census counts are made in December in each of these years it is reasonable to regard the figures as an expression of the mean population for 1930/31, 1946/47 (the same figures used by Olinder (1953) in the Swedish investigation) and 1950/51. We have calculated the figures for cancer ventriculi mortality for these periods with the result shown in Table I.

The figures for 1946/47 agree completely with those of Olinder (1953). They indicate a tendency toward lower cancer ventriculi mortality in the cities than in the country. For women between the ages of 40 and 70 there is even a decided difference between the mortality in the cities and in the country. The excess mortality for women of this age is just as pronounced in 1930/31. The same age

TABLE I.—*Number of Deaths from Cancer Ventriculi per year per* 100,000 *of the Native Population in Norway.*

| Age in years. | 1930/31. Country. M. | F. | Cities. M. | F. | 1946/47. Country. M. | F. | Cities. M. | F. | 1950/51. Country. M. | F. | Cities. M. | F. |
|---|---|---|---|---|---|---|---|---|---|---|---|---|
| Under 30 | 0·1 | 0·6 | 0·3 | — | 0·3 | 0·2 | — | — | — | 0·2 | 0·2 | — |
| 30–39 | 8 | 7 | 10 | 3 | 6 | 3 | 6 | 7 | 5 | 4 | 4 | 4 |
| 40–49 | 43 | 33 | 36 | 21 | 20 | 18 | 25 | 16 | 21 | 16 | 23 | 14 |
| 50–59 | 126 | 86 | 137 | 51 | 78 | 49 | 77 | 45 | 75 | 40 | 75 | 37 |
| 60–69 | 328 | 224 | 342 | 179 | 233 | 138 | 228 | 99 | 186 | 119 | 185 | 115 |
| 70 and over | 559 | 425 | 595 | 488 | 551 | 383 | 542 | 497 | 555 | 404 | 537 | 296 |
| Total | 64 | 53 | 60 | 47 | 57 | 44 | 56 | 42 | 56 | 44 | 55 | 37 |

groups in 1950/51 show little difference between cities and country, but in contrast to 1930/31 and 1946/47 there is an excess mortality for the country population in the age-group 70 and over.

As regards cancer ventriculi mortality for men, there is no tendency toward a systematic difference between cities and country. What can be concluded is that the mortality is not significantly higher in the cities than in the country for any of the two-year periods as it is for the other forms of cancer.*

There does not seem to be any systematic change in the relationship between cancer ventriculi mortality in the cities and in the country in the course of the period 1930/31–1950/51. According to the medical statistics there has been a very considerable reduction in cancer ventriculi mortality during the 20-year period in both the cities and the country. This is illustrated in Table II.

TABLE II.—*Percentage Reduction in Cancer Ventriculi Mortality from* 1930/31–1950/51.

| Age. | Country. M. | F. | Cities. M. | F. |
|---|---|---|---|---|
| 50–59 | 40·5 | 57·5 | 45·3 | 27·5 |
| 60–69 | 43·3 | 46·9 | 45·9 | 35·8 |
| 70 and over | 0·7 | 4·9 | 9·7 | 39·3 |

It seems that cancer ventriculi mortality in the age-groups between 50 and 70 was about 40 per cent lower in 1950/51 than in 1930/31, while the reduction is more uneven for the ages over 70. For most of the groups the reduction

* The death rates per 100,000 population in 1950–51 for cancer of the stomach in England and Wales (calculated from data given in the Registrar-General's Statistical Reviews for those years) were :

| | Males. 25– | 45– | 65– | 75+ | Females. 25– | 45– | 65– | 75+ |
|---|---|---|---|---|---|---|---|---|
| Conurbations | 6 | 63 | 213 | 293 | 4 | 31 | 127 | 244 |
| Smaller urban areas | 6 | 65 | 216 | 304 | 3 | 29 | 125 | 240 |
| Rural districts | 4 | 53 | 191 | 287 | 3 | 27 | 113 | 230 |

has taken place equally rapidly from 1946/47 to 1950/51 as from 1930/31 to 1946/47, so there is every reason to believe that cancer ventriculi mortality will decrease still more.

In other words the statistics indicate clearly that the city environment affects cancer ventriculi in a different manner from other types of cancer. According to the statistics this is apparently true; but statistics are never better than the material on which they are based; and diagnosis may be a weak point in the mortality statistics. In the above mentioned publication from Stockholm's Bureau of Statistics it is stated that the distribution of deaths among causes of death is not exact for the country districts. There is reason to believe that the diagnoses are not quite exact in the cities either, but in Denmark and Norway also they are probably more correct in the cities than in the country.

We shall return later to the possibility that incorrect diagnosis may be a significant cause of the statistically lower mortality from cancer ventriculi in the cities. For the present we use the Swedish calculations as statistical indications that this is a real situation, or, as Olinder states: " It is obvious that the strength and nature of the factors which further or inhibit the disease are essentially different in the cities and in the country districts." But he adds: " Obviously, a more accurate grouping of the population is necessary in order to determine a causal connection in the question of the origin of the disease."

Our problem is really a particularization of the Swedish calculations and is based on Stocks' (1950) results from England: Can it be demonstrated that mortality from cancer ventriculi differs among various population groups in Oslo?

It appears that cancer ventriculi is a special case as regards the relationship between mortality in the cities and in the country. It is highest in the country, while the reverse is true of most other causes of death. On the other hand, Stocks' (1950) figures indicated that cancer ventriculi, like most other causes of death, takes its heaviest toll of the poorest level of the population. The apparent conflict between these two statements makes it difficult to determine what factors are at work—and what, for example, is the situation for different parts of Oslo.

Primarily with the idea of examining this latter question we have made a special compilation of all deaths from cancer ventriculi in Oslo (without Aker) from 1930–50 from the records of the Oslo Bureau of Public Health and from death certificates. We shall first examine the figures for the individual age groups for the population of Oslo as a whole (Table III).

TABLE III.—*Deaths from Cancer Ventriculi in Oslo (without Aker)* 1930–50.

| | Men. | | | Women. | | |
|---|---|---|---|---|---|---|
| Age. | Number of deaths. | Native population December 1, 1937. | Deaths per 100,000 population per year 1930–50. | Number of deaths. | Native population December 1, 1937. | Deaths per 100,000 population per year 1930–50. |
| Under 20 | 2 | — | — | 0 | — | — |
| 20–29 | 2 | 26,319 | 0·4 | 3 | 31,439 | 0·5 |
| 30–39 | 20 | 24,754 | 4 | 25 | 30,469 | 4 |
| 40–49 | 117 | 19,383 | 29 | 82 | 23,234 | 17 |
| 50–59 | 255 | 12,094 | 100 | 165 | 16,705 | 47 |
| 60–69 | 452 | 8,391 | 257 | 369 | 12,425 | 141 |
| 70–79 | 384 | 3,801 | 481 | 454 | 6,951 | 311 |
| 80 and over | 135 | 870 | 739 | 279 | 2,028 | 655 |

During the course of the 21-year period, 1930–50, mortality from cancer ventriculi has decreased. It is, therefore, not surprising that cancer ventriculi mortality for the period as a whole is somewhat higher than the corresponding figures in the Swedish calculations for 1946/47. However, the relationship between the age-groups and the proportional mortality for men and women remains about the same.

We have calculated cancer ventriculi mortality for the east and west sides of the city separately. The division has been made by parishes as follows :

West side parishes :

Vår Frelser

Trefoldighet

Frogner

Uranienborg

Fagerborg

Gamle Aker

Markus

East side parishes :

Jakob

Sagene

Torshov

Paulus

Petrus

Grønland

Kampen

Gamlebyen

Vålerenga

Table IV shows mortality for the populations of the two parts of the city.

TABLE IV.—*Cancer Ventriculi Mortality on the East and West Sides of Oslo*, 1930–50.

Deaths per year per 100,000 population (12/1–1937).

| | West side. | | | East side. | | | Entire city. | | |
|---|---|---|---|---|---|---|---|---|---|
| Age. | M. | F. | Total. | M. | F. | Total. | M. | F. | Total. |
| 50–59 | 70 | 39 | 51 | 127 | 56 | 88 | 100 | 47 | 69 |
| 60–69 | 198 | 103 | 139 | 305 | 186 | 239 | 257 | 141 | 188 |
| 70–79 | 456 | 261 | 324 | 503 | 371 | 422 | 481 | 311 | 371 |

For the present we shall consider only the typical cancer ventriculi age groups. The age groups 50–79 years included more than ¾ of all deaths from cancer ventriculi in Oslo during the period 1930–50.

The figures reveal at once that, if the material, *i.e.* the diagnosis, is correct, cancer ventriculi mortality between the ages of 50 and 80 is decidedly higher on the east side of the city than on the west. For each of the three age-groups and for both sexes it is obvious that the mortality figures for the west side are lower and those for the east side higher than the corresponding figures for the city as a whole. The difference in cancer ventriculi mortality between the west and east sides is more than 50 per cent for the two youngest age-groups, for the group 70–79 years the difference is not quite as marked.

If we study the figures more closely we find that the difference between cancer ventriculi mortality on the west and east sides of the city is somewhat greater for men than for women in the age-group 50–59 years, but considerably greater for women than for men in the age-groups 60–69 and 70–79 years.

There are no census figures for each separate year as regards age distribution in the various parishes. For this reason we have used the municipal census figures of December 1, 1937 as a basis for calculating mortality. It is the census which is closest to the middle of the period 1930–50. In order to determine whether chance variations in the sex distribution play an important part, we have also calculated the mortality on the basis of an average of the age distributions on December 1, 1930, December 1, 1937, and October 1, 1948. This gives the figures in Table V.

TABLE V.—*Mortality from Cancer Ventriculi on the West and East Sides of Oslo*, 1930–50.

Deaths per year per 100,000 population 12/1 1930–12/1 1937–10/1 1948.

| | West side. | | | East side. | | | Entire city. | | |
|---|---|---|---|---|---|---|---|---|---|
| Age. | M. | F. | Total. | M. | F. | Total. | M. | F. | Total. |
| 50–59 | 64 | 37 | 48 | 108 | 49 | 76 | 88 | 43 | 62 |
| 60–69 | 201 | 102 | 138 | 299 | 178 | 230 | 253 | 137 | 184 |
| 70–79 | 431 | 243 | 302 | 456 | 350 | 392 | 445 | 291 | 346 |

It is found that the mortality figures on the average are slightly lower when based on this average age distribution than when the age distribution on December 1, 1937, is used as a basis. However, the reduction affects the east and west side figures to about the same extent and therefore does not lead to any shift in their mutual relationship.

The mortality figures for the other age groups of significance for cancer ventriculi, 30–49 years and over 80, do not change the impression that cancer ventriculi mortality is higher on the east than on the west side of the city (Table VI).

TABLE VI.—*Mortality from Cancer Ventriculi on the West and East Sides of Oslo*, 1930–50.

Deaths per year per 100,000 population December 1, 1937.

| | West side. | | | East side. | | | Entire city. | | |
|---|---|---|---|---|---|---|---|---|---|
| Age. | M. | F. | Total. | M. | F. | Total. | M. | F. | Total. |
| 30–49 | 11 | 7 | 9 | 16 | 13 | 14 | 14 | 10 | 12 |
| 80 and over | 696 | 527 | 568 | 775 | 837 | 813 | 731 | 655 | 680 |

Here it is clear that the difference between the east and west sides is relatively moderate for men, but very considerable for women. In the age-group 30–49 years the mortality is almost twice as high for women on the east side as on the west. It is characteristic that for both this age-group and for the group 80 years and over the mortality for men on the west side is lower than that for women on the east side, and on the east side the mortality for women is even higher than that for men in the oldest age-group. In other words it seems that on the east side women have been exposed to the irritants which may be responsible for higher mortality more than men, but that this difference is more pronounced for the oldest age-groups.

The object of dividing the Oslo population into east and west sides of the city was to obtain a population group with a generally good standard of living and one with a somewhat lower standard. There can, undoubtedly, be some question as to whether our division is the correct one. It is quite superficial. It can be demonstrated that income per consumer's unit as well as housing conditions were, on the average, better in that part of the city which we have called " west side " than in the part we have called " east side," but this does not afford a really sensitive expression for the relationship between cancer ventriculi mortality and the social level in the period 1930–50 in Oslo.

The mortality figures for the various parishes will give a more differentiated picture (Table VII).

TABLE VII.—*Annual Mortality: Number of Deaths per* 100,000 *Population on December* 1, 1937, *from Cancer Ventriculi for the Age-groups* 50–79 *Years in the Period* 1930–50 *in the Individual Parishes of Oslo without Aker.*

| Parish. | Men. | Women. | Total. |
|---|---|---|---|
| West side: | | | |
| Vår Frelser | 167 | 110 | 134 |
| Trefoldighet | 177 | 117 | 140 |
| Frogner | 190 | 89 | 126 |
| Uranienborg | 152 | 103 | 120 |
| Fagerborg | 142 | 100 | 115 |
| Gamle Aker | 193 | 136 | 157 |
| Markus | 227 | 87 | 136 |
| East side: | | | |
| Jakob | 218 | 149 | 180 |
| Sagene | 223 | 147 | 180 |
| Torshov | 253 | 116 | 178 |
| Paulus | 229 | 141 | 177 |
| Petrus | 235 | 161 | 192 |
| Grönland | 293 | 207 | 247 |
| Kampen | 292 | 182 | 230 |
| Gamlebyen | 255 | 155 | 198 |
| Vålerenga | 214 | 174 | 192 |
| Oslo total | 214 | 130 | 164 |

In this table it is readily seen that the figures are relatively low for the most typical west side parishes and relatively high for the south-eastern parishes, and the tendency is convincingly obvious in the cartogram which has been made on the basis of these figures (Fig. 1). However, we still lack a more concrete characterization of the "social level" of the individual parishes. A characterization of this type would have to have a large number of components in order to be complete. But when only one single factor is used—and that should be permissible in this case—the average values for the amount paid in rent should probably be the most reliable.

On the basis of the municipal statistics of December 1, 1936, we have calculated weighted averages for seven groups of parishes. In order to obtain figures which are comparable with the figures for cancer ventriculi mortality, we had to make this grouping instead of giving the figures for each individual parish (Table VIII).

The negative correlation between cancer ventriculi mortality and average annual rent is not absolute in the sense that the mortality is highest in the group of parishes where the average annual rent is lowest, next highest in the group where the rent is next lowest, etc. The reason why the correlation is not complete may also be that rent is not an accurate characterization of living standards. At least there is no doubt that there is a negative correlation. We have drawn up the points in a diagram (Fig. 2) and indicated the curve which seems to illustrate the relationship between cancer ventriculi mortality and the social standard—characterized by the annual rent level—in each group of parishes. With so few points we have not calculated the mathematical line

FIG. 1.—Annual mortality of cancer of the stomach—number of deaths per 100,000 population —for the age group 50–79 years. The period 1930–50 in Oslo.

of regression. As the figures stand the correlation does not follow a straight line, and the curve which is indicated is probably closer to the actual situation.

The conclusion again is that the material indicates that cancer ventriculi mortality on the average is higher among the poorer classes of the population in Oslo than among those better off. Before discussing the possible causes, on the assumption that the conclusion has a basis in fact, we shall examine in more

TABLE VIII.—*Annual Cancer Ventriculi Mortality for the Age-groups* 50–79 *Years in Seven Groups of Parishes and Annual Average House Rent in the same Groups of Parishes.*

| Parishes. | Cancer ventriculi mortality. Men. | Women. | Total. | Average annual rent in 1936. |
|---|---|---|---|---|
| Våar Frelser | 167 | 110 | 134 | 905 Crowns |
| Trefoldighet, Markus, Gamle Aker | 197 | 115 | 145 | 923 ,, |
| Frogner, Uranienborg, Fagerborg | 165 | 96 | 120 | 1,290 ,, |
| Sagene and Torshov | 236 | 134 | 179 | 631 ,, |
| Paulus | 229 | 141 | 177 | 534 ,, |
| Petrus | 235 | 161 | 192 | 567 ,, |
| Grønland, Kampen, Gamlebyen, Jakob, Vålerenga | 260 | 178 | 215 | 543 ,, |
| Whole city | 214 | 130 | 164 | 765 ,, |

FIG. 2.—The relationship between cancer ventriculi mortality and the average house rent in Oslo 1930–50. The annual number of deaths from cancer of the stomach per 100,000 population for the age group 50–79 years.

detail the possible statistical errors that were mentioned above. Can incorrect diagnosis of cancer ventriculi have occurred systematically both in the country districts and in Oslo? Let us concentrate on Oslo to determine whether the statistics of the Bureau of Health can throw any light on this question.

According to the statistics of the Oslo Bureau of Health, a little more than 37 per cent of all cancer deaths in Oslo in 1930 were due to cancer of the stomach. In 1949 the corresponding figure was 22 per cent. Part of the explanation of the relative decrease in cancer ventriculi mortality as compared to total cancer mortality may be that cases which would previously have been recorded in the group which has always been the largest, namely cancer ventriculi, would now be recorded as cancer in other organs according to the more accurate present diagnostics.

Cancer of the liver, for example, may have been confused with cancer of the stomach. This would also explain why the difference between cancer ventriculi mortality in the cities and in the country districts is smaller in Sweden than in Norway and Denmark. According to Swedish practice, the nomenclature used in that country classifies cancer in both the stomach and the liver in the same group, so that an exact distinction between deaths from these two forms of cancer in the cities and in the country would not be so marked in the total statistics.

We have reviewed the statistics from the Oslo Bureau of Health and have found no marked tendency as regards the ratio between the number of deaths from cancer in the liver and gall bladder (cancer in these two organs was recorded in the same group until 1951 in the mortality statistics of the Bureau of Health) and cancer of the stomach in the period 1930–50. The number of deaths from cancer in the liver and gall bladder comprise between 15 and 25 per cent (in one year, 1943, almost 30 per cent) of the number of deaths from cancer ventriculi, and even though there is a slight tendency toward an increase in the later part of the period the difference is too small to influence the proportion of cancer ventriculi mortality to total cancer mortality.

The number of deaths from cancer of the oesophagus is of a magnitude similar to deaths from cancer of the liver and gall bladder, but no definite tendency can be detected for the development of this form of cancer in the period 1930–50 either.

On the other hand, mortality from cancer of the colon shows a very characteristic tendency in the period 1930–50. If we divide the 21 years into 5-year periods, excluding 1950, the figures found are shown in Table IX.

TABLE IX.—*Mortality in Oslo (without Aker), 1930–50. (Figures from Oslo Bureau of Health.)*

| Period. | Cancer ventriculi. 1. | Cancer coli. 2. | 2 as a percentage of 1. |
|---|---|---|---|
| 1930–34 | 686 | 100 | 14·6 |
| 1935–39 | 715 | 153 | 21·4 |
| 1940–44 | 668 | 175 | 26·2 |
| 1945–49 | 658 | 232 | 35·3 |
| 1930–49 | 2,727 | 660 | 24·2 |

Here it is clearly seen that the number of deaths from cancer coli, in contrast to the number from cancer ventriculi, has increased considerably during the period 1930–50, and while the number of deaths from the former was only 14·6 per cent of the latter in 1930–34, it had increased to 35·3 per cent in 1945–49.

Nevertheless, the sum of deaths from these two forms of cancer has comprised a constantly decreasing proportion of total deaths from cancer during the period 1930–50. The increase in the number of deaths from cancer coli has not been sufficient to compensate the decrease in deaths from cancer ventriculi. The sum of the number of deaths from cancer of the stomach and of the colon represented not quite 35 per cent of all deaths from cancer during 1930–49, but the percentage proportion decreased from almost 39 in 1930–34 to about 33·5 in 1945–49.

Looking at the mortality from cancer coli (the statistics do not distinguish between cancer of the large and small intestine, but the majority of the cases are cancer of the large intestine, excluding rectum), we arrive at the figures in Table X.

TABLE X.—*Number of Deaths from Cancer Coli per 100,000 Living per year in Norway.*

| Age-group. | Males. 1930–31. | Males. 1950–51. | Females. 1930–31. | Females. 1950–51. |
|---|---|---|---|---|
| 30–39 | 2 | 1 | 1 | 0·5 |
| 40–49 | 3 | 4 | 4 | 3·0 |
| 50–59 | 11 | 12 | 17 | 14·0 |
| 60–69 | 39 | 29 | 25 | 29·0 |
| 70–79 | 52 | 85 | 60 | 84·0 |
| 80 years and more | 65 | 164 | 45 | 207·0 |

It is easy to notice the marked rise in mortality for the age group above 70, and there is no doubt about the significant increase of cancer coli as cause of death in general occuring in the oldest group of the population. The corresponding figures for Oslo shows a similar picture (Table XI).

TABLE XI.—*Number of Deaths from Cancer Coli per* 100,000 *Living per Year in Oslo.*

| | Males. | | Females. | |
|---|---|---|---|---|
| Age-group. | 1930–31. | 1950–51. | 1930–31. | 1950–51. |
| 30–39 | 7 | 3 | — | 1 |
| 40–49 | — | 6 | 6 | 4 |
| 50–59 | 4 | 18 | 19 | 15 |
| 60–69 | 66 | 47 | 37 | 32 |
| 70–79 | 63 | 121 | 58 | 99 |
| 80 years and more | 77 | 205 | 59 | 247 |

Without a thorough revision of the material, that is, death certificates, charts, etc., it will be difficult to know if the statistics here express a true picture of the reality. It is of some interest to compare the development registered in the statistics of Norway during the period 1930 to 1950 to similar figures in England and Wales (Table XII).

TABLE XII.—*Death Rates per* 100,000 *Population in* 1931–35 *and* 1950–51 *for Cancer of the Stomach and Colon in England and Wales* (*Calculated from the Registrar-General's Statistical Reviews*).

| | Stomach. | | | | Large intestine.* | | | |
|---|---|---|---|---|---|---|---|---|
| | Males. | | Females. | | Males. | | Females. | |
| Age-group. | 1931–35. | 1950–51. | 1931–35. | 1950–51. | 1931–35. | 1950–51. | 1931–35. | 1950–51. |
| 35 | 12 | 9 | 7 | 5 | 4 | 5 | 6 | 5 |
| 45 | 42 | 36 | 23 | 16 | 16 | 12 | 18 | 16 |
| 55 | 107 | 98 | 64 | 45 | 52 | 37 | 49 | 39 |
| 65 | 219 | 210 | 151 | 124 | 138 | 114 | 126 | 94 |
| 75+ | 262 | 296 | 221 | 239 | 224 | 235 | 239 | 229 |

* Excluding rectum.

It is at once apparent that the tendency which appears so clearly in the Norwegian figures—fall in the mortality in cancer ventriculi and rise in the mortality from cancer of the large intestine—is not present in the same manner in England and Wales. In England and Wales, on the contrary, a fall in the mortality both from cancer ventriculi and coli in the period 1931 to 1951 is found, except in the age group 75+.

On the whole it would appear that improved diagnostic methods can hardly be the whole explanation of the decreasing significance of cancer ventriculi as a cause of death as compared to other forms of cancer in Oslo. If the figures represent a true tendency, it might be that the primary cause is to be found in the improvement in the standard of living which has undoubtedly taken place during the period 1930–50. If we assume that cancer ventriculi, more than other forms of cancer, is dependent on the " standard of living," the relative (and also absolute)

decrease in cancer ventriculi mortality is plausible. And it is in good agreement with the finding that cancer ventriculi mortality is higher on the east side than on the west.

In this connection it is interesting to notice that mortality from cancer coli—according to the information afforded from the statistics of the Oslo Bureau of Health—tends to be higher on the west side than on the east. When all deaths from cancer coli in the individual parishes in Oslo during 1930–50 are considered in relation to the native population aged 50 and over on December 1, 1937, we find 56 deaths per 100,000 on the west side as compared to 47 per 100,000 on the east side.

A statistical demonstration of the variation in cancer ventriculi mortality for the two parts of the city during the period 1930–50 would be desirable. Unfortunately our material does not provide figures which would make this possible and the mortality statistics of the Oslo Bureau of Health do not give sufficiently specified figures for such a calculation. Nevertheless, the tendency in development should appear from the following rather summary frequencies : Total number of deaths from cancer ventriculi (according to the Oslo Bureau of Health) in each part of the city in relation to the native population over 40 years of age in the respective parts of the city. The figures are shown in Table XIII.

TABLE XIII.—*Approximate Annual Mortality from Cancer Ventriculi for the Population of Oslo Aged* 40 *Years or More.*

| | West side. | | | East side. | | | Entire city. | | |
|---|---|---|---|---|---|---|---|---|---|
| Period. | M. | F. | Total. | M. | F. | Total. | M. | F. | Total. |
| 1930–34 | 144 | 111 | 124 | 206 | 147 | 174 | 178 | 128 | 149 |
| 1935–39 | 130 | 109 | 17 | 181 | 129 | 153 | 158 | 118 | 135 |
| 1940–44 | 125 | 77 | 95 | 146 | 117 | 130 | 137 | 97 | 113 |
| 1945–49 | 103 | 67 | 81 | 128 | 100 | 113 | 117 | 83 | 97 |
| Per cent decrease in mortality, 1930–34 to 1945–49 | 28·5 | 39·6 | 34·7 | 36·9 | 32·0 | 35·1 | 34·3 | 35·2 | 34·9 |

According to our hypothesis that the reduction in cancer ventriculi mortality is related to the improvement in living standard, one would expect that cancer ventriculi mortality would have decreased most markedly on the east side because it is reasonable to suppose that the population of the east side has benefited most from the general social improvement. But here again we know far too little of the changes in the consumer composition which have taken place in the various levels of the population. The propaganda for proper diet, for example, may have had just as much influence among the more well-to-do classes as among the poorer ones. At any rate it appears that the decrease in cancer ventriculi mortality has occurred almost parallel on the east and west sides of Oslo.

Our material may indicate that cancer ventriculi mortality varies with the standard of living, but the figures tell us nothing as to whether it is a lower standard of living in general—poorer housing, lower calorie consumption, harder work—which increases the disposition to cancer ventriculi, or whether one or several particular aspects of the lower standard of living are the decisive factors. The relation between cancer ventriculi mortality in the cities and in the country may lead us to believe that certain aspects of living conditions, which were common for both the country and the east side populations during 1930–50, are significant. In any case, examination of several details will be necessary to provide a clue as to some of the causes.

To find a reasonable starting point for such investigations, it is necessary to begin with guesses. We shall mention a few things which might prove profitable fields for further investigation.

*Housing conditions* were probably the factor which characterized the difference in living standard most markedly during this period for the various population groups. Housing may have a direct influence, both physical and mental, and bad housing may also be the cause of unsatisfactory personal hygiene and health care.

*Eating habits* undoubtedly vary with the economic level. The—unfortunately all too limited—household investigations which have been made in Norway afford some information. They indicate that poor economy often leads to little variation in the diet, often more prepared foods, lower vegetable consumption and perhaps higher consumption of salt and spiced foods.

Whatever line is followed, great difficulties will be encountered in obtaining the necessary data. As the statistical apparatus becomes better developed, for example the statistics which aim to record private consumption, the problem will be facilitated. And as the calculations of national income require such detail statistics there is hope of improvement along these lines. At any rate the Cancer Registry will provide invaluable assistance in obtaining reliable material on cancer. There should be no doubt that this will afford the basis for profitable research in future years.

### SUMMARY.

With a starting point in the hypothesis that cancer is environmentally determined we have compiled a material comprising deaths from cancer ventriculi in Oslo (without Aker) during the period 1930–50 from the records of the Oslo Bureau of Health.

The material seems to confirm that cancer ventriculi mortality in this period differed in various parts of the city and that it was, on the whole, higher in the eastern than in the western parts of Oslo. The relation between cancer ventriculi mortality and the economic level is also clear when the former is compared in relation to a single measure of the " social level " in seven groups of parishes in Oslo, namely the average annual house rent. The rule is that a high rent coincides with low cancer ventriculi mortality.

The figures show that cancer ventriculi mortality has decreased considerably from 1930 to 1950, this is true of the entire country, cities and country districts, and Oslo in particular.

On the basis of the figures for Oslo we discuss whether the decrease in mortality from cancer ventriculi as revealed by statistics can be due to a reduction in incorrect diagnosis during the course of the period, so that during the last part of the period, more than earlier, only the diagnostically reliable cases were included in the mortality statistics for cancer ventriculi. The Oslo Bureau of Health figures reveal a regular increase in both the absolute and relative number of deaths from cancer coli simultaneous with the decrease in cancer ventriculi mortality. However, the increase in the number of deaths from cancer coli is not sufficient to compensate for the reduction in cancer ventriculi mortality. The number of deaths from cancer in the oesophagus, liver and gall bladder reveal no systematic tendency during this period. A confusion of different forms of cancer thus does not seem to provide the entire explanation of the decrease in cancer ventriculi mortality revealed by the statistics.

A Swedish investigation (Olinder, 1953) indicated that cancer ventriculi mortality—in contrast to mortality from cancer in other organs—was higher in the country districts than in the cities. Olinder's figures for 1946/47 indicate that the tendency is the same in Norway. Norwegian figures for 1930/31 and 1950/51, however, do not afford a basis for the contention of a significant difference between cancer ventriculi mortality in cities and country districts. For most other causes of death the mortality is highest in the cities, so cancer ventriculi differs from the others in this respect.

If there is a real connection between cancer ventriculi mortality and social conditions it would be reasonable to expect that the reduction in cancer ventriculi mortality from 1930 to 1950 would be greater among the east side than among the west side population in Oslo. The available figures do not indicate that this is so ; the reduction has been almost parallel in both parts of the city.

Finally there is a discussion of the individual factors which might be responsible for the tendency to excess mortality from cancer ventriculi among the poorer classes of the population of Oslo. Housing conditions, hygiene, eating habits and diet may be significant. Further investigations of these factors are necessary, however, in order to obtain more exact knowledge of their importance.

### REFERENCES.

OLINDER, O.—(1953) Kanserdödeligheten i stadene och på landsbygden. Statistisk Månadsskrift för Stockholm stad nr. 10.

STOCKS, P.—(1950) *Brit. J. Cancer*, **4**, 147.

THONER, J.—(1924) Kræftsygdommene i Gol og Hemsedal 1902–21. Tillegg for Norsk Magasin for lægevidenskap.

WÅLER, G. H. M.—(1931) ' Über die Erblichkeit des Krebses.' Oslo (Dybwad).